# ON Bipolar Cells in Macaque Retina: Type-Specific Synaptic Connectivity with Special Reference to OFF Counterparts

**DOI:** 10.3389/fnana.2016.00104

**Published:** 2016-10-27

**Authors:** Yoshihiko Tsukamoto, Naoko Omi

**Affiliations:** ^1^Studio EM-Retina, SatonakaNishinomiya, Japan; ^2^Department of Biology, Hyogo College of MedicineNishinomiya, Japan

**Keywords:** monkey retina, ribbon synapse, cone photoreceptor, rod photoreceptor, retinal ganglion cell, vision, serial section electron microscopy, neural circuits

## Abstract

To date, 12 macaque bipolar cell types have been described. This list includes all morphology types first outlined by Polyak (1941) using the Golgi method in the primate retina and subsequently identified by other researchers using electron microscopy (EM) combined with the Golgi method, serial section transmission EM (SSTEM), and immunohistochemical imaging. We used SSTEM for the rod-dense perifoveal area of macaque retina, reconfirmed ON (cone) bipolar cells to be classified as invaginating midget bipolar (IMB), diffuse bipolar (DB)4, DB5, DB6, giant bipolar (GB), and blue bipolar (BB) types, and clarified their type-specific connectivity. DB4 cells made reciprocal synapses with a kind of ON-OFF lateral amacrine cell, similar to OFF DB2 cells. GB cells contacted rods and cones, similar to OFF DB3b cells. Retinal circuits formed by GB and DB3b cells are thought to substantiate the psychophysical finding of fast rod signals in mesopic vision. DB6 cell output synapses were directed to ON midget ganglion (MG) cells at 70% of ribbon contacts, similar to OFF DB1 cells that directed 60% of ribbon contacts to OFF MG cells. IMB cells contacted medium- or long-wavelength sensitive (M/L-) cones but not short-wavelength sensitive (S-) cones, while BB cells contacted S-cones but not M/L-cones. However, IMB and BB dendrites had similar morphological architectures, and a BB cell contacting a single S-cone resembled an IMB cell. Thus, both IMB and BB may be the ON bipolar counterparts of the OFF flat midget bipolar (FMB) type, likewise DB4 of DB2, DB5 of DB3a, DB6 of DB1, and GB of DB3b OFF bipolar type. The ON DB plus GB, and OFF DB cells predominantly contacted M/L-cones and their outputs were directed mainly to parasol ganglion (PG) cells but also moderately to MG cells. BB cells directed S-cone-driven outputs almost exclusively to small bistratified ganglion (SBG) cells. Some FMB cells predominantly contacted S-cones and their outputs were directed to OFF MG cells. Thus, two-step synaptic connections largely narrowed down the S-cone component to SBG and some OFF MG cells. The other OFF MG cells, ON MG cells, and ON and OFF PG cells constructed M/L-cone dominant pathways.

## Introduction

Vertebrate retina's bipolar cells terminate close to the photoreceptor layer with one polar neurite and to the ganglion cell layer with the other polar neurite. The bipolar cells play the central part of neuronal wirings. This observation helped substantiate Cajal's idea of the independent “neuron” as the primary structural and functional unit of nervous tissue (Cajal, 1893). Based on this notion, Polyak (1941) clarified the cellular organization of primate retina. He used the tissue stained by the Golgi method for light microscopy. Both authors, however, could not reveal the precise structure of cellular interfaces. EM ultimately revealed the synaptic cleft between two nerve cell membranes, confirming the neuron doctrine. Nevertheless, identification of individual synapses in randomly sampled sections does not reveal how neurons are wired into circuits. A precise description of neuronal circuitry at EM resolution requires high-quality SSTEM.

**Figure 1:**
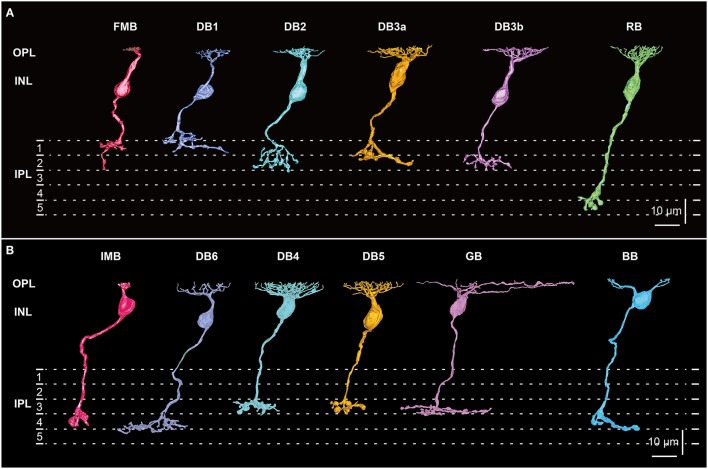
**Morphology and stratification of all 12 types of macaque bipolar cells**. **(A)** Five OFF cone bipolar types: flat midget bipolar (FMB), diffuse bipolar (DB) 1, 2, 3a, and 3b, and one ON rod bipolar (RB) type. **(B)** Six ON cone bipolar types: invaginating midget bipolar (IMB), diffuse bipolar (DB) 4, 5, and 6, giant bipolar (GB), and blue bipolar (BB). Each stratum of the IPL (1−5) is 6 μm thick. Strata 1−2 and 3−5 comprise the OFF and ON sublaminae, respectively.

Currently, there are 12 known bipolar cell types in macaque retina (Figure 1), 10 of which were successfully distinguished by combining Golgi staining with EM (Boycott and Dowling, 1969; Kolb et al., 1969; Kolb, 1970; Mariani, 1984; Boycott and Wässle, 1991; Boycott and Hopkins, 1993) and subsequently confirmed by immunohistochemical staining (Grünert et al., 1994; Haverkamp et al., 2003; Puthussery et al., 2011). The 11th type, the “giant bipolar (GB),” was identified by Joo et al. (2011) through extensive observation of Golgi-stained macaque retina. These authors suggested that GB cell dendrites may participate in cone-selective connections because a single GB cell contacted only about half of the cones in its dendritic field. However, a more precise characterization of GB cell connectivity is required to determine whether the sparse innervation of cones indeed confers specific chromatic signaling. The 12th bipolar cell type, DB3b, was found by two groups independently. Puthussery et al. (2013) identified DB3b as a novel type distinct from DB3a (formerly DB3) based on immunological and electrophysiological properties, while Tsukamoto and Omi (2013, 2014) identified DB3b based on EM showing basal contacts with both rods and cones. In mice, three OFF types (3a, 3b, and 4) and one ON type (7) contact both rods and cones (Mataruga et al., 2007; Tsukamoto et al., 2007; Haverkamp et al., 2008; Tsukamoto and Omi, 2014). It is thus of interest to determine if this is a specialization specific to species heavily reliant on dark light conditions or if any primate ON bipolar cells also contact rods and cones to form mixed pathways.

The bipolar cell ON and OFF distinction has been determined by the receptor proteins at the dendritic tips, mGluR6 for the ON types and iGluRs (AMPA and kainate) for the OFF types. Although, invaginating synapses are prevalent on ON bipolar dendrites, this positional class of synaptic contact is not essential for preserving ON polarity but rather is related to the efficacy of neurotransmitter diffusion (DeVries et al., 2006; Szmajda and DeVries, 2011). Several studies (Hopkins and Boycott, 1995, 1996, 1997; Calkins et al., 1996; Chun et al., 1996) have shown semi-invaginating (or triad-associated) synapses at ON bipolar dendrites. We previously analyzed the positional classes of basal synapses at OFF bipolar dendrites (Tsukamoto and Omi, 2015a,b). Here we attempt to extend the same analysis to ON bipolar cells for their further characterization.

S-cones predominantly contact BB cells (Mariani, 1984; Kouyama and Marshak, 1992). The connection of S-cones with other types of ON bipolar cells appears to be unfavorable but still occurs at low frequencies (Lee et al., 2004; Lee and Grünert, 2007). Whether S-cones contact any other ON bipolar cell types is critical for understanding chromatic processing. As a related circuital problem, about half the ribbon synapses of the OFF DB1 cell axon terminal are directed to OFF MG cells, so it is an interesting question whether any type of non-IMB ON bipolar cell directs output predominantly to ON MG cells.

Here, we reconstructed all types of ON (cone) bipolar cell in full for several cells and additionally a number of cells in part by SSTEM. The examination area was an ovoid perifoveal region of roughly 100 μm in the horizontal axis and 70 μm in the vertical axis. No unclassified bipolar cells were left within this area. We clarified their neuronal connections through synaptic contacts at the ultrastructural level to obtain specific structural evidence to answer our current questions.

## Materials and methods

### Macaque monkey retina

A series of 817 radial sections was prepared for SSTEM from the perifoveal region of the right retina of a 7-year-old female Japanese monkey (*Macaca fuscata*, 6.5 kg). This animal was kindly donated by the psychophysical research group in the (former) Electrotechnical Laboratory of the Ministry of International Trade and Industry, Japan. This SSTEM series is the same as that used in previous studies by Tsukamoto and Omi (2014, 2015a), where the details of sample preparation were described. The procedure was performed in compliance with the Guide for the Care and Use of Experimental Animals (Hyogo College of Medicine).

### Electron microscopy

Here, we briefly describe several key points of our procedures. After dual perfusion with aldehyde fixative via (post-vitrectomy in front of the fovea) intraocular and intravascular passages, tissue blocks of retina with intact sclera and choroid were isolated, post-fixed with a mixture of 2% osmium tetroxide and 1% potassium ferricyanide, and stained *en bloc* with 3% uranyl acetate in 80% methanol. Blocks were embedded in Araldite resin and cut in serial sections at a setting thickness of 90 nm using a Leica UCT ultramicrotome (Leica microsystems, Welzlar, Germany). Sections were mounted on 120 formvar-coated single-slot grids and stained with 3% uranyl acetate in 80% methanol and Reynolds' lead citrate. These staining protocols provided sufficient image contrast to discriminate fine cytological features.

Electron micrographs of the section series were acquired at both 400 × and 3000 × using a JEM 1220 electron microscope (Jeol Ltd., Tokyo, Japan) at the Joint-Use Research Facilities of Hyogo College of Medicine. Twenty-four overlapping negative images were acquired from each individual section at 3000 × to capture a 90 × 187 μm area covering the outer plexiform layer (OPL) to the ganglion cell layer in a 4 × 6 montage. These images were enlarged 4-fold; thus, the final magnification of prints used for image analysis was 12,000 ×. The examination area was located 3.00−3.25 mm temporal to the foveal center and the center of the examination area was approximately 15° from the foveal center. This area is characterized by highest rod density and the features of peripheral circuits.

We traced every neuronal process while marking synapses and other features with color pens on transparent sheets. The digitized contour lines were saved on a personal computer using Intuos-4 digitizer (Wacom, Saitama, Japan) and TRI/3D-SRF-R graphics software (Ratoc Systems International, Tokyo, Japan). For graphic representation of electron micrographs and reconstructed neuronal digital images, we used Photoshop and Illustrator in Adobe CS6 (Adobe Systems, San Jose, CA).

### Classification procedures

It is well known that S-cones can be distinguished from M/L-cones by their unique innervation of BB cells (Mariani, 1984; Kouyama and Marshak, 1992; Wässle et al., 1994). S-cone pedicles were also distinctly smaller in area and volume than M/L-cone pedicles (Kolb, 1991; Kolb and Dekorver, 1991). In this study, we found 35 BB cells connected to three (each partly included in the series) small bistratified ON-blue ganglion cells (Dacey and Lee, 1994; Calkins et al., 1998; Dacey et al., 2014). Using these BB connections, we identified 19 S-cones and employed 8 S-cones for detailed analysis. The density of S-cones was 1.2 × 10^3^ pedicles/mm^2^, whereas that of all cones was 12.6 × 10^3^ pedicles/mm^2^. Therefore 9.5% of the cones were of S-type in this examination area.

Three morphological variables at the level of light microscopy were used primarily for classification of mammalian bipolar cells, axon-to-ganglion cell layer (GCL) distance (the distance between the axon terminal tip and the border line of the IPL and GCL), stratification thickness of the axon arbor, and planer axon arbor area (e.g., Kolb et al., 1981; Cohen and Sterling, 1990; Boycott and Wässle, 1991; Euler and Wässle, 1995; Badea and Nathans, 2004; Ghosh et al., 2004; Li et al., 2004; Pignatelli and Strettoi, 2004). In accordance with these studies, we measured the same variables from three-dimensionally reconstructed bipolar cells. The definitions of these three variables were explained pictorially in our previous article (Figure 3 in Tsukamoto and Omi, 2014). In addition, we used ultrastructural variables of bipolar synaptic contacts with photoreceptors, PG cells, and MG cells at the electron microscopic level to distinguish bipolar cell types.

We used Statistica 06J (Statsoft Japan, Tokyo, Japan) for testing the distribution fit (chi-square test) of GB cell contacts with cones and for cluster analysis (Ward's joining method) to differentiate ON bipolar cell types. Quantitative data are presented as the mean ± standard deviation (SD) and number of samples (n) in tables and figures unless otherwise indicated.

### Cone-ganglion connection strength

We evaluated the cone sampling strength of a ganglion cell by the product of the synaptic contact numbers between cones and ON bipolar cells and between ON bipolar and midget ganglion cells. Each ganglion cell connects to several bipolar cells via a variable number of synaptic contacts, where *w*_*gi*_ is the number of contacts between the *g*-th ganglion and *i*-th bipolar cell (*i* = *1, 2*, · · ·, *m*). In turn, each bipolar cell connects to several cones via a variable number of synaptic contacts, where *v*_*ij*_ is the number of contacts between the *i*-th bipolar and *j*-th cone (*j* = *1, 2*, · · ·, *n*). The sum of the products of contact numbers *w*_*gi*_ *v*_*ij*_ for all *m* bipolar cells yields the connection strength between the *g*-th ganglion cell and *j*-th cone.

$$\begin{array}{l} p_{gj}=\sum_{i=1}^{m}w_{g_{i}}v_{ij}=w_{g_{1}}v_{1j}+w_{g_{2}}v_{2j}+\cdot \;\cdot \;\cdot \;+w_{g_{m}}v_{mj} \end{array}$$

The sum of these contact number products for all convergent cones yields an estimate of the total connection strength (*P*_*g*_) of the *g*-th ganglion cell with its cone field:

$$\begin{array}{l} P_{g}=\sum_{j=1}^{n}p_{gj}=p_{g1}+p_{g2}+\cdot \;\cdot \;\cdot \;+p_{gn}. \end{array}$$

This formulation is widely used as a model of three-layer networks (Jordan, 1986).

## Results

### Twelve types of macaque bipolar cells

All 12 types of macaque bipolar cells, each in side-view, are shown in Figure 1. The dendrites of all bipolar types stratify at the same level in the OPL, but the axons terminate type-dependently in different strata (1~5) of the inner plexiform layer (IPL). One aim of this study is to find similarities between ON and OFF bipolar cell types. We represent possible corresponding ON and OFF pairs in the same color. Reasons for these pairings are described in detail in the Discussion. Since we have already described five OFF types (FMB, DB1, 2, 3a, and 3b) of (cone) bipolar cells in detail (Tsukamoto and Omi, 2014, 2015a), here we focus on six ON types (IMB, BB, DB4, DB5, DB6, and GB) of (cone) bipolar cells. Although, a rod bipolar (RB) cell is presented for comparison with cone bipolar cells, the RB type was not examined further in this study.

Each IMB cell extended a single dendritic process, with the terminal arbor spanning a single cone pedicle (less than 10 μm). In contrast, the BB cells extended 1~3 dendritic processes, each with a terminal arbor spanning a single cone pedicle. The BB cells extending a single dendritic process were similar in shape to IMB cells, although the BB axon terminal arbor was slightly larger than that of the IMB counterpart. The DB4 cells extended a short dendrite stout with a densely branching arbor. By contrast, DB6 cells extended a thinner primary dendrite with a sparsely branching arbor. The thickness of the DB5 cell dendrite appeared intermediate between those of DB4 and DB6 cells. The dendritic arbor of a GB cell was markedly wide. The RB cell had mop-like dendrites composed of many fine processes.

The axon terminals of OFF bipolar cells are located in strata 1 and 2 of the IPL, whereas those of ON bipolar cells are in strata 3, 4, and 5 of the IPL. The DB4, DB5, and GB axon terminals examined here were confined to stratum 3 close to the OFF-ON border, while the IMB axon terminals extended over stratum 4, and the DB6 and BB axon terminals were located across stratum 4 and the upper half of stratum 5. The RB axon terminals reached the deepest part of stratum 5. Next, we examined the synaptic ultrastructure of each ON bipolar cell type.

### Dendritic and axonal synapses of ON cone bipolar cells

**Figure 2:**
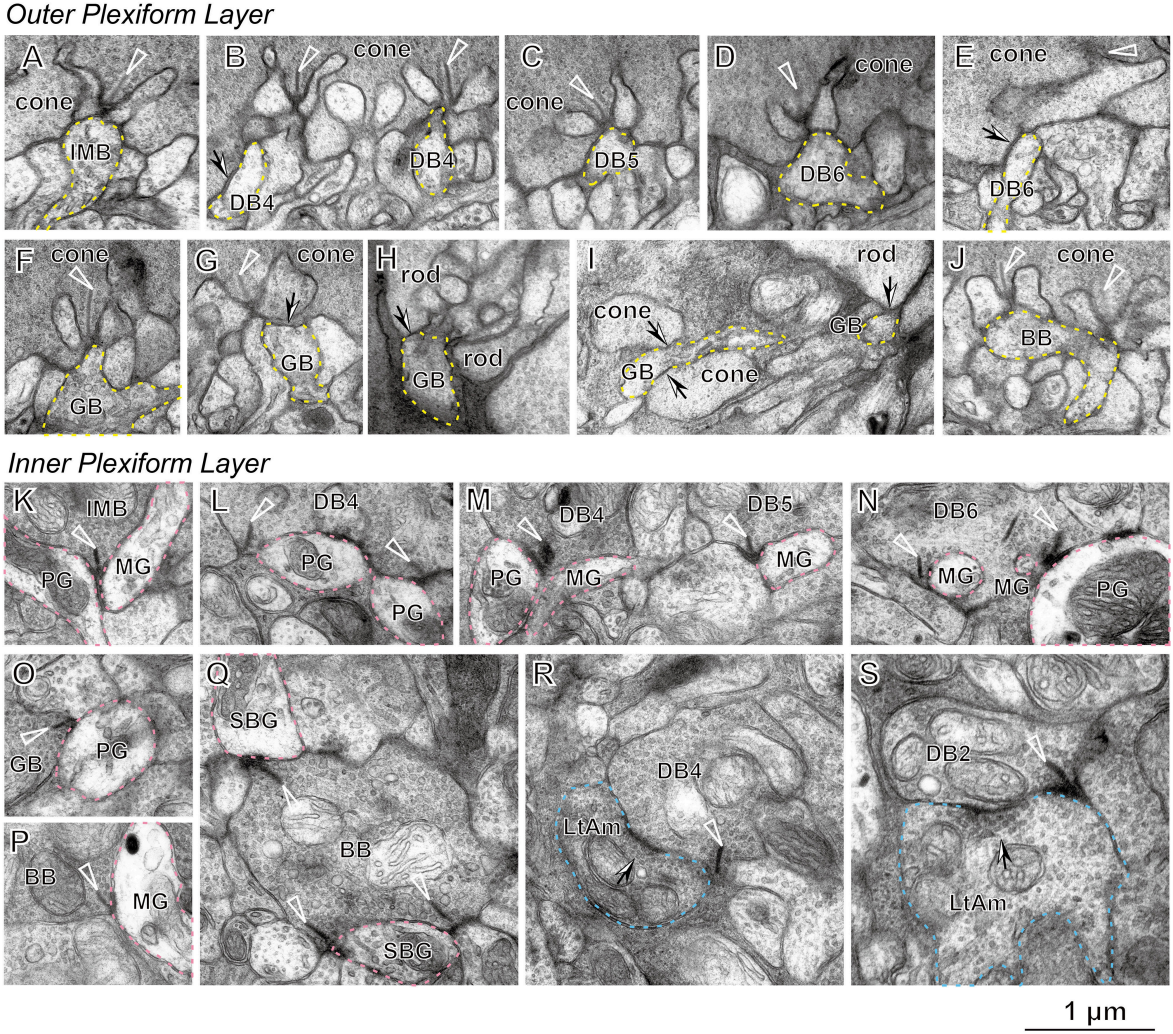
**Electron micrographs of bipolar cell synapses with cone and rod photoreceptors (A−J), parasol and midget ganglion cells (K−Q), and ON-OFF lateral amacrine cells (R,S)**. An invaginating dendrite (dotted yellow contour) of the bipolar cell makes a synaptic contact with the cone pedicle under the ribbon (arrowhead) for IMB **(A)**, DB4 (**B**, right), DB5 **(C)**, DB6 **(D)**, GB **(F)**, and BB **(J)**. A TA contact (arrow) of the bipolar dendrite (dotted yellow contour) with the cone pedicle is seen for DB4 (**B**, left), DB6 **(E)**, and GB6 **(G)**. NTA contacts (arrows) of the GB dendrite are also seen with two cone pedicles (**I**, left). TA contacts of the GB dendrite (dotted yellow contour) are found with rod spherules **(H)** and (**I**, right). Synaptic contacts associated with the ribbon of bipolar axon terminals (arrowhead) to ganglion cell dendrites (dotted pink contour) are seen for IMB to PG and MG **(K)**, DB4 to PG **(L)**, DB4 to PG and MG, and DB5 to MG **(M)**, DB6 to PG and MG **(N)**, GB to PG **(O)**, BB to MG **(P)**, and BB to SBG **(Q)**. An ON-OFF lateral amacrine cell (dotted light blue contour) has a reciprocal synapse (arrow) with DB4 **(R)** and DB2 **(S)** in association with the ribbon (arrowhead). PG, parasol ganglion cell; MG, midget ganglion cell; SBG, small bistratified ganglion cell; and LtAm, ON-OFF lateral amacrine cell.

Figure 2 presents typical electron micrographs of synaptic contacts at the dendritic tips and axonal terminals of the ON bipolar cells used in the following analyses. There were three positional classes of cone−bipolar contacts. A triad is comprised of one invaginating bipolar cell dendrite and two lateral horizontal cell processes just underneath the presynaptic ribbon in the basal cavity of a cone pedicle. We defined an invaginating contact as a synaptic contact between the invaginating dendrite and the pedicle base. All six types of ON bipolar cells exhibited invaginating contacts where the postsynaptic bipolar dendrites fully invaginated into the basal folds of the cone pedicle and contacted the presynaptic membrane in apposition to the cytoplasmic ribbon (Figures 2A–D,F,J). Most of the contacts made by IMB and BB cells were of this invaginating class. In contrast, DB and GB cells frequently exhibited the triad-associated (TA) or also-called semi-invaginating contact, defined as a contact between the pedicle base and the bipolar dendrite adjacent to a full-invaginating dendrite (Figures 2B,E,G). These DB and GB cells also displayed the non-triad-associated (NTA) contact, defined as a contact between the pedicle base and the bipolar dendrite that was separated by two or more processes (invaginating and/or TA processes) from the ribbon zone (Figure 2I). Most frequently, DB6 cells had TA contacts and GB cells had NTA contacts. Furthermore, GB cell dendrites occasionally contacted the basal membrane of rod spherules (Figures 2H,I). These rod contacts were always separated from the ribbon zone by the full- or semi-invaginating dendrites of rod bipolar cells.

To characterize the synaptic contacts at the axon terminals of ON bipolar cells, we examined two representative types of ON ganglion cells, a parasol ganglion (PG) and a midget ganglion (MG) cell, and also one unique amacrine cell type that made synaptic contacts in both ON and OFF sublaminae. All types of ON cone bipolar cells other than BB exhibited ribbon-associated synaptic contacts of variable frequency with both ON PG and MG cells (Figures 2K–O). In contrast, BB cells almost always had ribbon-associated synaptic contacts with small bistratified ganglion cells (Figure 2P), but only rarely with ON MG cells (Figure 2Q) and never with ON PG cells.

The ON-OFF amacrine cell projected thin lateral dendrites in all radial directions and made reciprocal synapses with DB4 cells (Figure 2R) as well as with DB2 cells (Figure 2S). We defined a reciprocal synapse as an amacrine return synapse with spacing less than half of a micron from a nearby bipolar ribbon synapse. When we annotated the bipolar axon arbor processes, these reciprocal synapses were critically important for distinguishing DB4 from DB5 processes (intermixing of dendritic arbors in the OPL and the axon terminals at the same strata of the IPL) and DB2 from DB3b processes (Tsukamoto and Omi, 2015a). This amacrine cell, which we call an ON-OFF lateral amacrine cell, thus acts as a criterion neuron for classification of bipolar cells and so is first described in detail.

### ON-OFF lateral amacrine cell as a criterion neuron for ON bipolar cell classification

**Figure 3:**
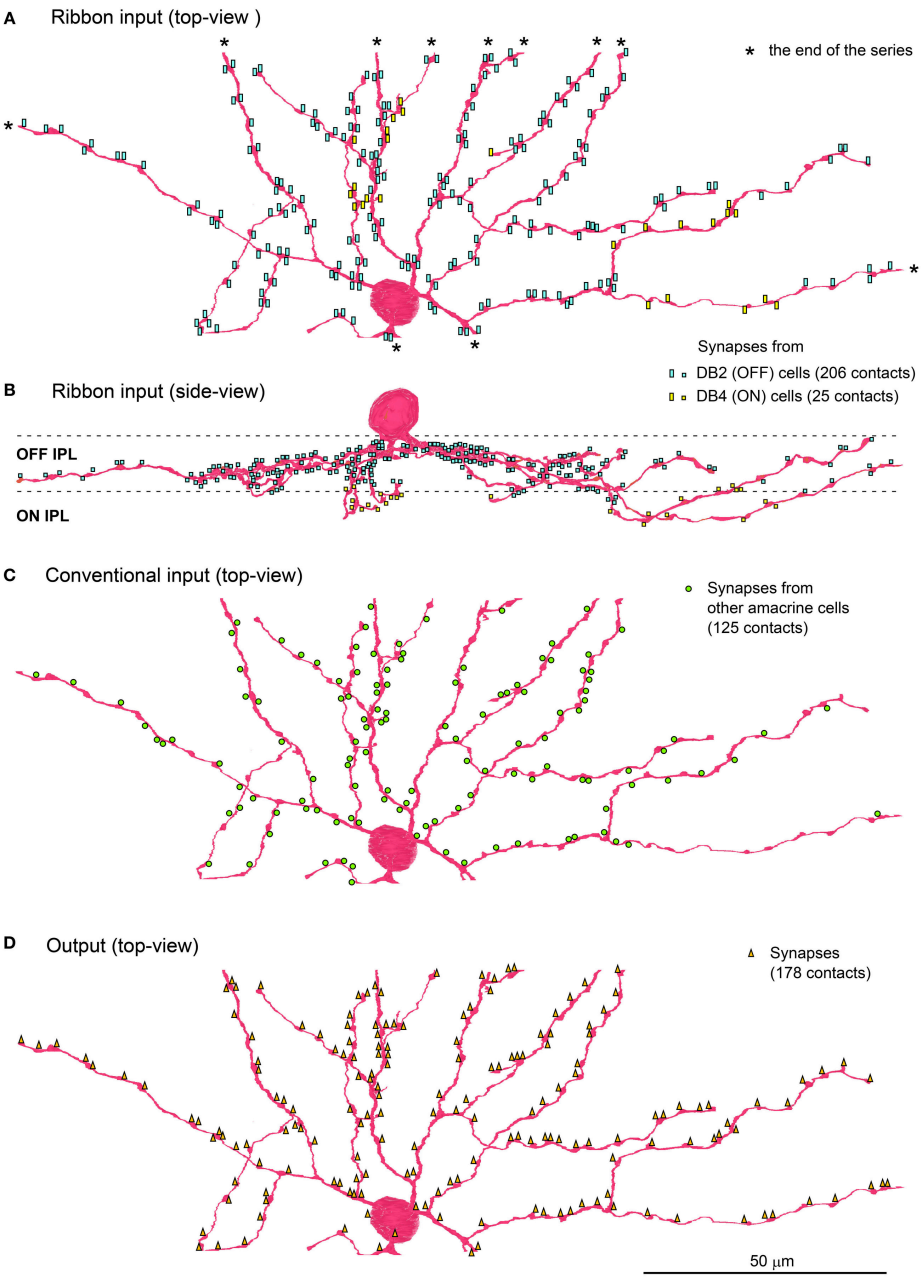
**ON-OFF lateral amacrine cell in contact with DB2 and DB4 cells**. Distribution of the postsynaptic sites of the ON-OFF lateral amacrine cell in contact with the ribbon-associated presynaptic sites of DB2 and DB4 cells is displayed in top view **(A)** and side view **(B)**. The contacts with DB2 (206 contacts labeled with light blue rectangles) are confined to strata 1 and 2 of IPL while those with DB4 (25 yellow rectangles) are confined to stratum 3. **(C)** Conventional input synapses (125 green circles) from other amacrine cells. **(D)** Output synapses (178 orange triangles) are mostly reciprocally directed to DB2 (146 contacts) and DB4 (24 contacts) cells and less often to other cells. Asterisks (^*^) indicate the end points of the series of electron micrographs.

The soma and part of the dendritic arbor of an ON-OFF lateral amacrine cell were contained in this series. The thin dendrites ran radially with branching as seen in the top view and passed through the upper IPL (strata 1 and 2) down to the lower IPL (strata 3) as seen in the side view (Figures 3A,B). Both input and output synapses were evenly distributed over the entire dendrite, although approximately 90% were in the OFF sublamina and 10% were in the ON sublamina. The postsynaptic sites for input were divided into two classes by the synaptic features, ribbon-associated vesicle clusters in the bipolar axon terminals (231 sites, Figures 3A,B) for one class and conventional vesicle clusters in the amacrine cell dendrites (125 sites, Figure 3C) for the other. The presynaptic sites for output (178 sites in total) were divided into three classes by the postsynaptic cell type, bipolar (172 sites), amacrine (5 sites), and ganglion (1 site) (Figure 3D).

The postsynaptic sites receiving the ribbon input from DB2 cells (206 sites), DB4 cells (24 sites), and (rarely) other cells (1 site from DB6 and 1 site from RB) were accompanied by reciprocal output sites with spacing of less than half a micron. Although, the return output sites to DB2 cells (146) were shorter than the DB2 ribbon input sites, about 60 output sites came in close proximity to two ribbon input sites, providing both with reciprocal synapses. The return output sites to DB4 (24) were as numerous as the DB4 ribbon input sites (25). Thus, 231 ribbon input sites were accompanied by 227 reciprocal output sites, so 98% of the ribbon synapses were reciprocal.

These observations suggest some functional significance of the lateral interaction with both DB2 and DB4 cells, which resembles the lateral inhibition conferred by the reciprocal feedback of horizontal cell dendrites to the ribbon synapses at photoreceptor terminals. The ON-OFF lateral amacrine cell also resembles the A17 amacrine cell in that both types have reciprocal synapses with bipolar cells, the former with DB2 and DB4 cells and the latter with rod bipolar cells (Nelson and Kolb, 1985; Hartveit, 1999). Interestingly, Elgueta et al. (2015) demonstrated that the GABA release from A17 cells to RB cells is modulated by acetylcholine released from starburst amacrine cells.

### Distribution of three classes of synaptic contacts with cones and rods

**Figure 4:**
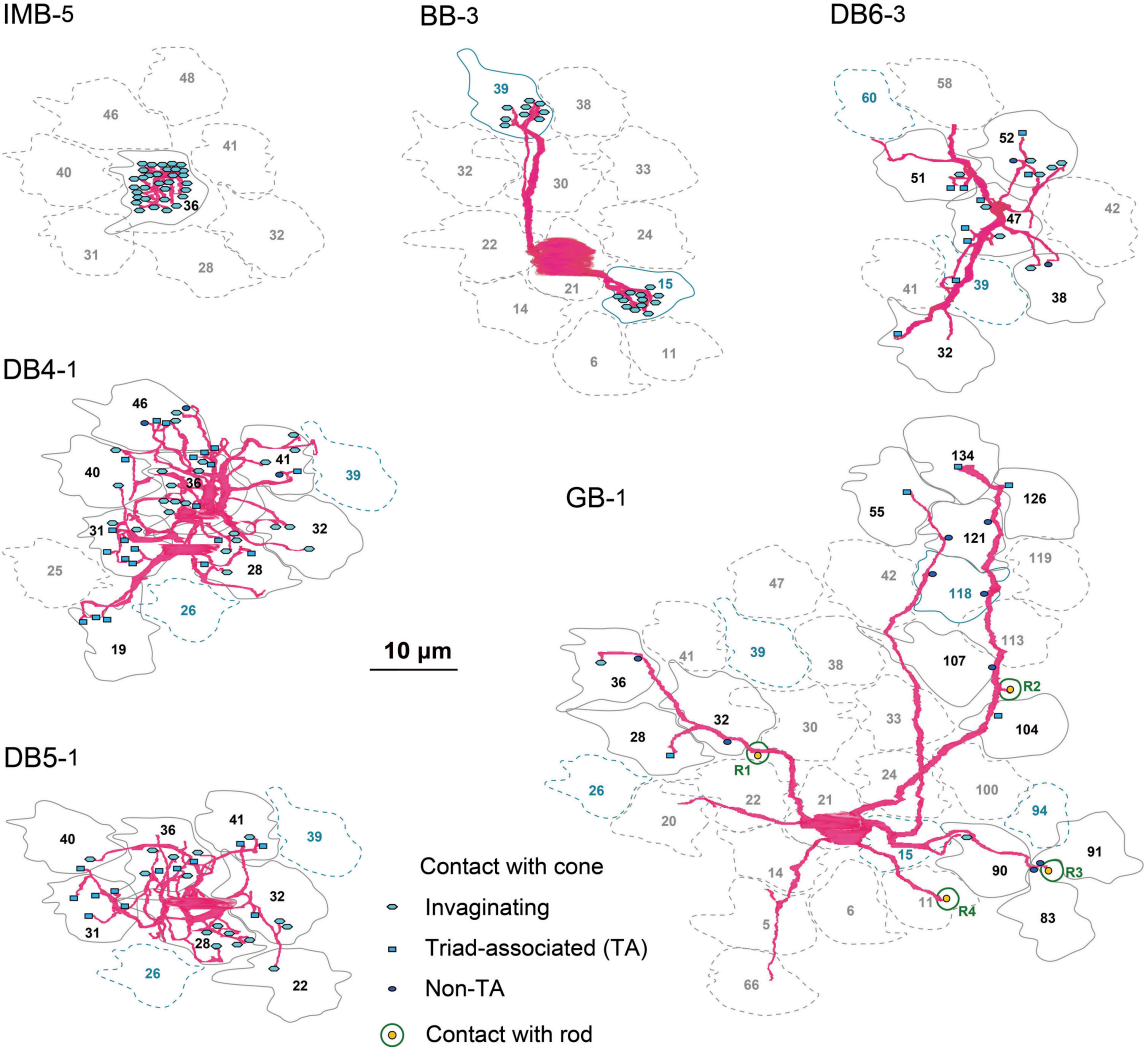
**Dendritic innervation of cone pedicles by the three positional classes of synaptic contacts for the six types of ON bipolar cells**. Cone pedicles are labeled by serial numbers and accordingly in the following figures. The cellular contours are shown with continuous lines for innervated cones and dotted lines for non-innervated cones. M/L-cone contours are colored gray and S-cone contours blue.

**Figure 5:**
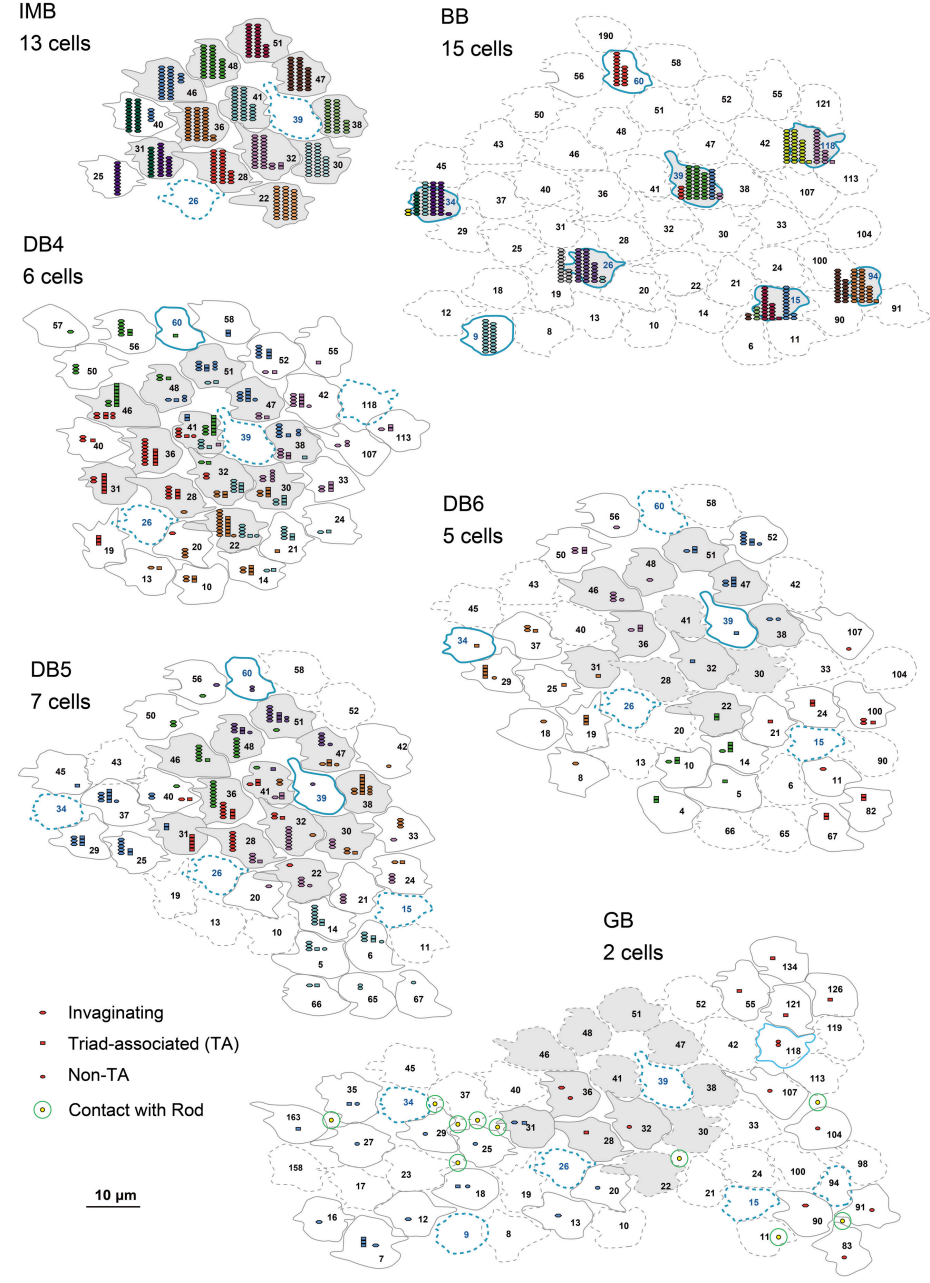
**Abundance of the three positional classes of synaptic contacts over the entire cone area for each ON bipolar cell type**. A group of ON bipolar cells of the same type shows a characteristic distribution of contacts with cone pedicles. Three positional classes of contacts with cones are invaginating (hexagons), TA (rectangles), and NTA (ellipses). Contacts with rods are only noted for GB. Twelve pedicles (22, 28, 30, 31, 32, 36, 38, 41, 46, 47, 48, and 51) for IMB, DB, and GB, and six S-cone pedicles (15, 26, 34, 39, 94, and 118) for BB, designated in gray, are used for contact analysis per cone in Figures 6D,E.

**Figure 6:**
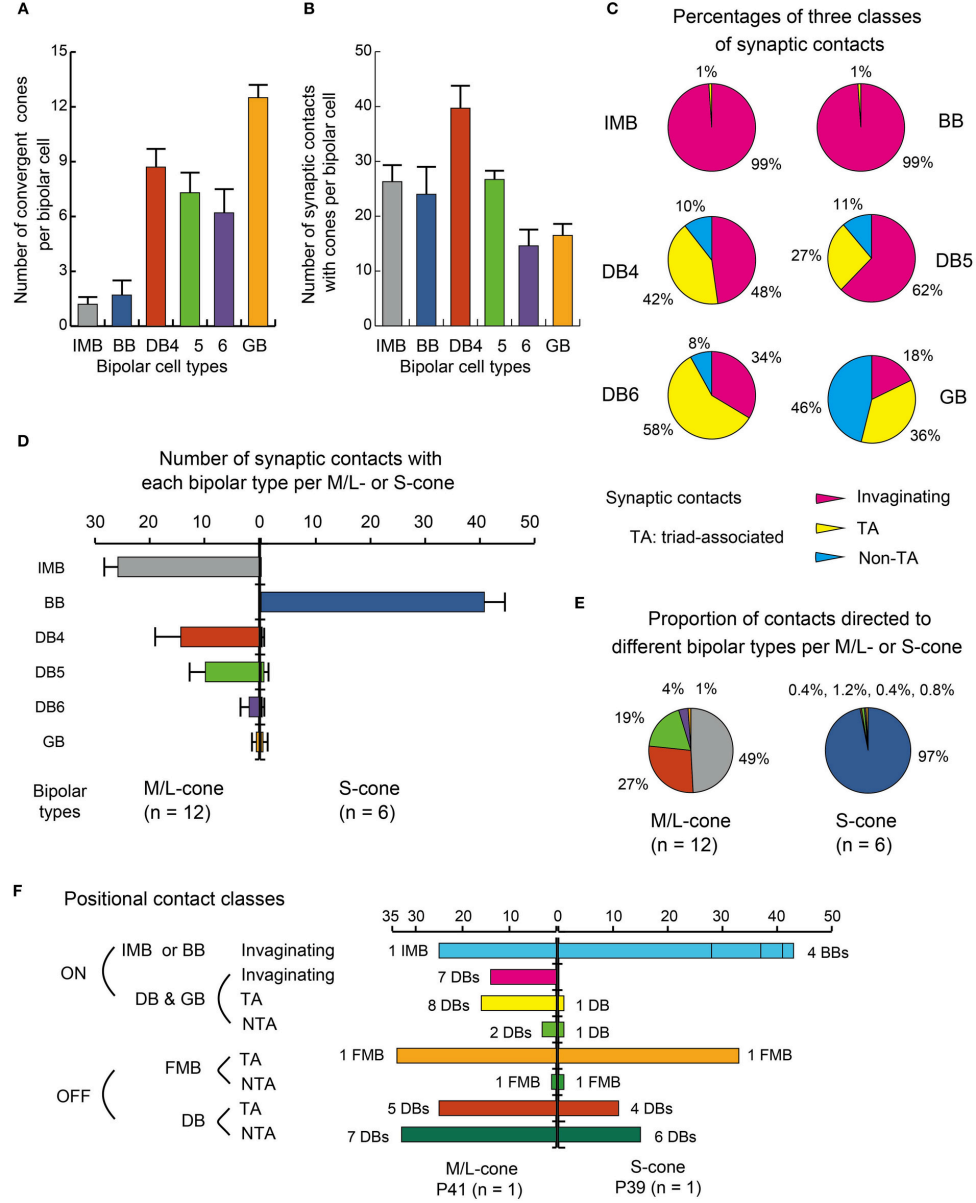
**Summary of dendritic synapse measurement. (A)** The number of cones converging onto each bipolar cell (every cone was equally counted as unity regardless of the number of contacts). **(B)** The total number of contacts of the three positional classes between bipolar dendrites and cone pedicles. **(C)** Pie charts showing the average proportions of the three positional classes of synapses. Data are mean ± SD for each bipolar cell type (number of cells): IMB (6), BB (7), DB4 (6), DB5 (7), DB6 (5), GB (2) with SD **(A**−**C)**. **(D)** A comparison of M/L-cone (left-handed bars, *n* = 12) and S-cone (right-handed bars, *n* = 6) divergence for the six types of ON bipolar cells. The abscissa is the average number of contacts of a cone with each type of bipolar cells with the SD bar. **(E)** Pie charts comparing M/L-cones and S-cones in the innervation of different bipolar types. **(F)** Three positional classes of contacts: invaginationg, TA, and NTA in the ON and OFF routes diverging from an M/L-cone pedicle (P41) and an S-cone pedicle (P39) both located in the center of the examination area (Figure 5).

Here we characterize ON bipolar cells in terms of their dendritic synaptic contacts with cone pedicles. The distribution profile of three positional classes of contacts, invaginating, TA, and NTA, differed among types. Figure 4 displays individual contacts on the dendritic arbors of each type of bipolar cell, Figure 5 shows the numbers of classified contacts for several bipolar cells of each type on pedicle maps, and Figure 6 provides a graphical representation of the quantitative relationships. The exact quantitative descriptions are given in Table 1.

**Table 1:** **Synaptic convergence of cones onto each ON bipolar cell type**.

**Bipolar cell type (n) Number of cells**	**Number of cones per bipolar**	**Number of contacts per bipolar**					
		**All**	**Positional classes**			**Chromatic classes**	
			**Invagi**.	**TA**	**NTA**	**M/L-cone**	**S-cone**
IMB (12)	1.2 ± 0.4	26.3 ± 2.5	26.0 ± 2.6	0.3 ± 0.6	0.0 ± 0.0	26.5 ± 3.1	0.0 ± 0.0
BB (7)	1.7 ± 0.8	24.1 ± 4.8	23.9 ± 5.0	0.3 ± 0.5	0.0 ± 0.0	0.0 ± 0.0	24.1 ± 4.8
DB4 (6)	8.7 ± 1.0	39.7 ± 4.1	19.0 ± 3.3	16.7 ± 2.6	4.0 ± 2.8	39.5 ± 4.2	0.2 ± 0.4
DB5 (7)	7.3 ± 1.1	26.7 ± 1.6	16.7 ± 5.8	7.1 ± 4.3	2.7 ± 2.9	26.1 ± 2.1	0.6 ± 1.5
DB6 (5)	6.2 ± 1.3	14.6 ± 2.9	5.0 ± 2.8	8.4 ± 2.7	1.2 ± 0.8	14.2 ± 2.4	0.4 ± 0.5
GB (2)	12.5 ± 0.7	16.5 ± 2.1	3.0 ± 1.4	6.0 ± 1.4	7.5 ± 0.7	15.5 ± 3.5	1 ± 1.4

IMB cells most frequently connected to only one cone and only rarely to two cones with 22−29 contacts in total. In the latter cases, there were major and minor clusters of contacts (e.g., IMB-3 having 24 contacts with P46 and 4 contacts with P40, Figure 5). In contrast, the seven BB cells in our current sample connected to a variable number of cones: one cone in 3 cases, two cones in 3 cases, and three cones in 1 case. When contacting to more than one cone, a total of 17−31 contacts were allocated to those cones. For example, BB-2 has 28 contacts with P39 only, but BB-6 has 19 contacts with P9, 10 contacts with P34, and 2 contacts with P15. The dendritic profile of a BB cell contacting only one cone resembled that of the typical IMB cell, although the BB axon terminals were slightly more extended horizontally than those of IMB cells as described later. The dendritic areas were similar in size among DB4, 5, and 6, but the dendritic patterns were distinct. Specifically, the dendritic processes were dense for DB4, moderate for DB5, and sparse for DB6. Accordingly, DB4 contacted 8−10 cones, DB5 7−9 cones, and DB6 5−8 cones. Also, consistent with the differences in dendritic process density, the number of contacts on these dendrites was great for DB4 (37−48), intermediate for DB5 (24−29), and relatively low for DB6 (12−19). The dendritic area of GB cells was largest among all bipolar types, but the dendritic processes were sparse and devoid of innervation from many nearby cone pedicles. The total number of contacts was small for GB cells (15−18), whereas the number of connected cones was large (12−13). Consequently, a GB cell connected to any individual cone through only one or two contacts. Also, the GB cell had unique synaptic contacts with the basal surface of rod spherules, although the number of such rod contacts was moderate; GB-1 had contacts with 4 rods and 13 cones, and GB-2 had contacts with 6 rods and 12 cones (Figures 4, 5).

These ON bipolar types also exhibited differences in the positional classes of synaptic contacts with cones. Invaginating and TA contacts were generally located in the central area of the pedicle base, whereas most NTA contacts were in the marginal area. The dendritic processes of both IMB and BB cells were almost always (99%) fully invaginating into the basal cavity of cone pedicles, while TA contacts was scarce (1%) and no NTA contacts were observed. DB4, 5, 6, and GB cells expressed all classes of contacts but with different frequency distributions. Among these four types, the descending rank order frequency of invaginating contacts was DB4 (19) > DB5 (17) > DB6 (5) > GB (3). The total number of non-invaginating contacts (TA + NTA) was also highest in DB4 (21) but similarly abundant in the others (10 for DB5, 10 for DB6, and 14 for GB) (Table 1). Relative to total contacts, however, the most common type of contact for DB5 cells was invaginating (62%), for DB6 cells was TA (58%), and for GB cells was NTA (46%) (Figures 5, 6C).

These different ON bipolar cells also displayed distinct connectivity patterns to M/L- and S-cones, suggesting type-specific differences in chromaticity. IMB cells contacted only M/L-cones and BB cells contacted only S-cones. Thus, the M/L-cone−IMB pathway and the S-cone−BB pathway appear structurally independent. Other DB and GB cells almost exclusively contacted M/L-cones and only rarely contacted S-cones. Only one of 6 DB4 cells connected to one S-cone with one contact, two of 7 DB5 cells connected to two S-cones with one contact and two contacts, respectively, two of 5 DB6 cells connected to two S-cones each with one contact, and one of 2 GB cells connected to one S-cone with two contacts. Thus, the mean number of contacts with S-cones per bipolar cell (Table 1) was at most one. Additionally, these S-cone contacts with DB and GB cells were always located at non-invaginating positions (TA or NTA).

In Figure 5, 12 M/L-cones and 6 S-cones shown in gray were used for analysis of cone divergence, determined by identifying all partner bipolar cells together with the number of output synaptic contacts. We investigated all ON bipolar dendrites surrounding these target cones. The divergence patterns differed greatly between M/L- and S-cones (Table 2, Figures 6D,E). The M/L-cone directed almost half of all output synapses (26) to IMB cells and allocated the other half to four non-IMB cells in decreasing order DB4 (14) > DB5 (10) > DB6 (2) > GB (0.5). In contrast, the S-cones directed almost all outputs (40) to BB cells and sporadically allocated the few remaining contacts (1) to four other ON bipolar types. Thus, the mean number of DB and GB cells per S-cone and the mean number of contacts innervating on those DB or GB cells per S-cone were both at most 0.5 (Table 2).

**Table 2:** **Synaptic divergence of M/L- or S-cones to different ON bipolar cell types**.

**Bipolar cell type**	**Number of bipolar cells per cone**		**Number of contacts per cone**	
	**M/L-cone**	**S-cone**	**M/L-cone**	**S-cone**
IMB	1.1 ± 0.3	0.0 ± 0.0	25.8 ± 2.5	0.0 ± 0.0
BB	0.0 ± 0.0	3.2 ± 1.0	0.0 ± 0.0	40.8 ± 3.9
DB4	2.7 ± 1.1	0.2 ± 0.4	14.3 ± 4.7	0.2 ± 0.4
DB5	2.3 ± 1.0	0.3 ± 0.5	9.8 ± 2.9	0.5 ± 0.8
DB6	0.8 ± 0.5	0.2 ± 0.4	1.8 ± 1.6	0.2 ± 0.4
GB	0.3 ± 0.5	0.2 ± 0.4	0.5 ± 0.8	0.3 ± 0.8
Total	7.1 ± 1.7	4.0 ± 1.7	52.3 ± 5.9	42.0 ± 4.6

Number of sample cones is 12 for M/L and 6 for S.

Figure 6F shows the divergence of output from the representative M/L-cone and S-cone to their ON and OFF routes via three positional classes of contacts. M/L-cone pedicle P41 had 32 ribbons, and S-cone pedicle P39 had 34 ribbons. Each number of ribbons is close to its mean value at 3 mm eccentricity (Mean ± SD: 31.3 ± 3.4 M/L-cone, *n* = 23; 31.8 ± 2.8 for S-cone, *n* = 10, Tsukamoto and Omi, 2015a).

One IMB and 7 DB cells had a total of 39 invaginating contacts with P41, whereas 4 BB cells had 43 invaginating contacts with P39. Thus the invaginating contacts outnumbered the ribbons by 7~9. This was because one invagination housed two or more ON bipolar cell dendrites underneath a number of relatively long ribbons. Thus, all the central places of the invaginations formed by the basal membrane were occupied by IMB and some ON DB cell processes. Consequently, the other ON DB bipolar cell processes were situated at the adjacent places or more distant places. In M/L-ON system, we found 16 TA contacts and 3 NTA contacts. Taken together, roughly two thirds were invaginating and one thirds was basal (TA and NTA). In S-ON system, however, the other ON DB cells had distinctly small number of contacts. We found 1 TA contact and 1 NTA contact in the S-cone pedicle. Thus, 96% of the ON contacts were invaginating.

In M/L-OFF system, 1 FMB cell had 34 TA contacts and 1 NTA contact. OFF DB cells had 25 TA contacts and 33 NTA contacts. Thus all contacts were basal. In S-OFF system, 1 FMB cell had 33 TA contact and 1 NTA contact. OFF DB cells had 11 TA contact and 15 NTA contacts. The positional class profiles of DB types are similar between ML- and S-OFF systems. However, the total number of contacts was considerably smaller in the S-cone (105) than the M/L-cone (151) in accordance with the narrower area of the S-cone pedicle base.

### Statistics of dendritic synapses and cluster analysis

GB cells contacted only about half the cone pedicles in their dendritic area (Figure 5). This scarcity of cone−GB contacts suggests the possibility of cone-selective connections (Joo et al., 2011). To test whether the connection pattern is random or specifically targeted, we examined the distribution pattern. For cell GB-1, the frequency distribution of cones having the number (in round brackets) of contacts per cone regardless of positional class was 18 (0), 11 (1), and 2 (2). Likewise, cell GB-2 had the distribution: 9(0), 8(1), 3(2), and 1(4). These distributions were well fitted by the Poisson distribution with lambda values of 0.4 for GB-1 (χ^2^ = 0.55, *p* = 0.46, *n* = 31) and 0.8 for GB-2 (χ^2^ = 3.38, *p* = 0.34, *n* = 21), suggesting that cone−GB contacts are determined by random chance of rare events. There are at least two possible causes for this randomness, M-cones and L-cones are randomly intermingled or GB cell dendrites randomly contact M/L-cones indiscriminately, but these alternatives cannot be distinguished by this analysis.

**Figure 7:**
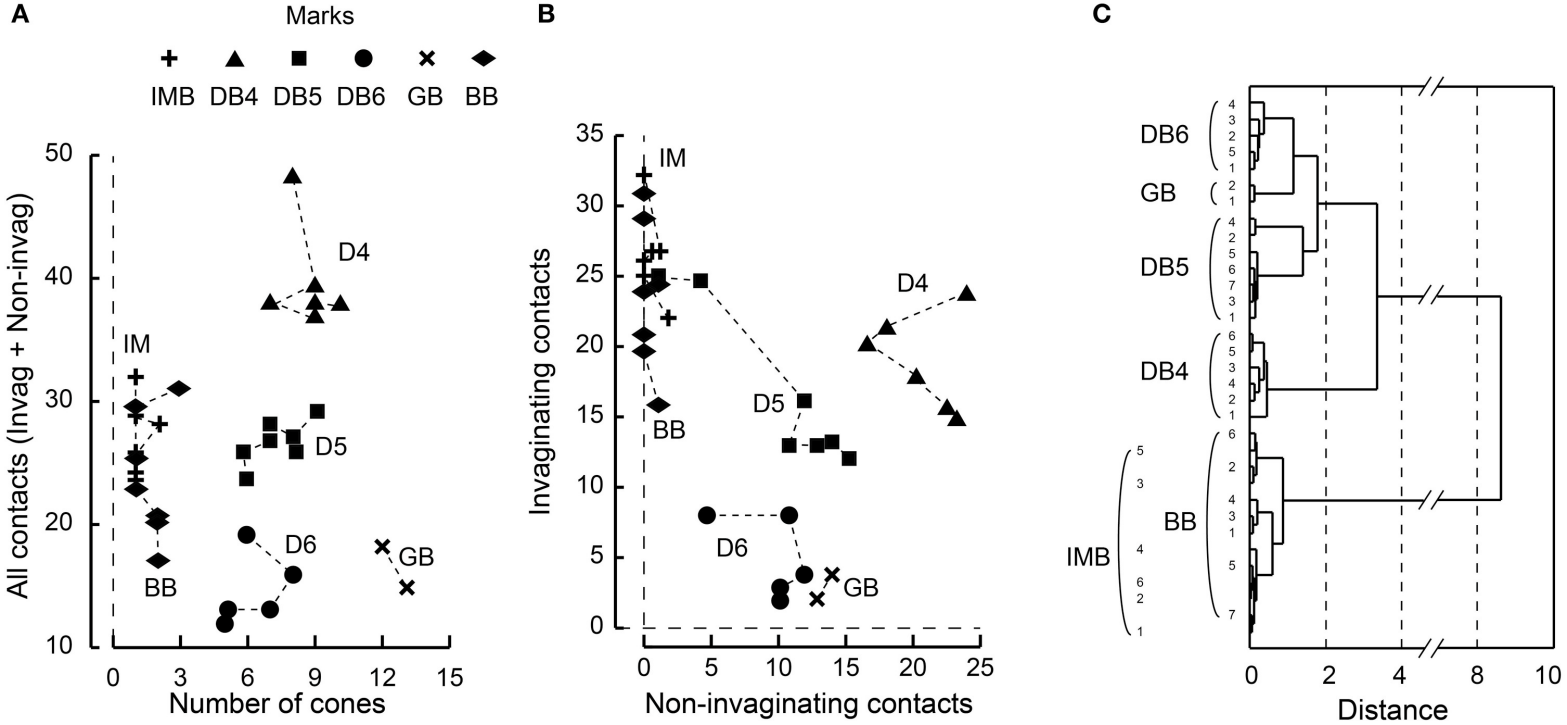
**Scatter plots of cone−bipolar contact counts and their clustering among the six ON bipolar cell types. (A)** The relationship between the total number of contacts (all classes) and the number of convergent cones per bipolar cell. **(B)** The relationship between the number of invaginating contacts and the number of TA and NTA contacts. **(C)** Dendrogram of cluster analysis (Ward's method) of 33 ON bipolar cells using the four variables plotted in **(A,B)**. DB4, DB5, DB6, and GB types are differentiated but IMB and BB are not.

Each ON bipolar cell type had a distinct distribution pattern of synaptic contacts on its dendrites. In fact, each type occupies its own unique area within the scatter plot for the total number of synaptic contacts (regardless of positional class) vs. the number of convergent cones (Figure 7A). DB4, 5, and 6 types are aligned on the cone number axis but separated from each other on the total contact number axis. IMB and BB types are separated from the other four types but not from each other on this plane. (Here we used only 6 IMB cells for analysis although we examined 12 IMB cells as shown in Table 1). The GB type is separated from the other five types on the cone number axis. To examine the discrimination power of the proportion of invaginating contacts, we plotted the number of invaginating contacts vs. the number of non-invaginating contacts (Figure 7B). Again DB4, 5, 6, and GB cells were separated to various extents, but IMB and BB cells were not separated from each other. When we divided the non-invaginating contacts into TA and NTA for analysis, GB was more distinctly separated from the others (not shown).

We applied cluster analysis to all 33 ON bipolar cells using the four variables described (Figure 7C). Types DB4, 5, 6, and GB were uniquely clustered but both IMB and BB were within the same cluster. This indicates that the dendritic synapses of IMB and BB have similar architectural characteristics.

### Morphology of bipolar cell axon arbors and connection to ganglion cells

**Figure 8:**
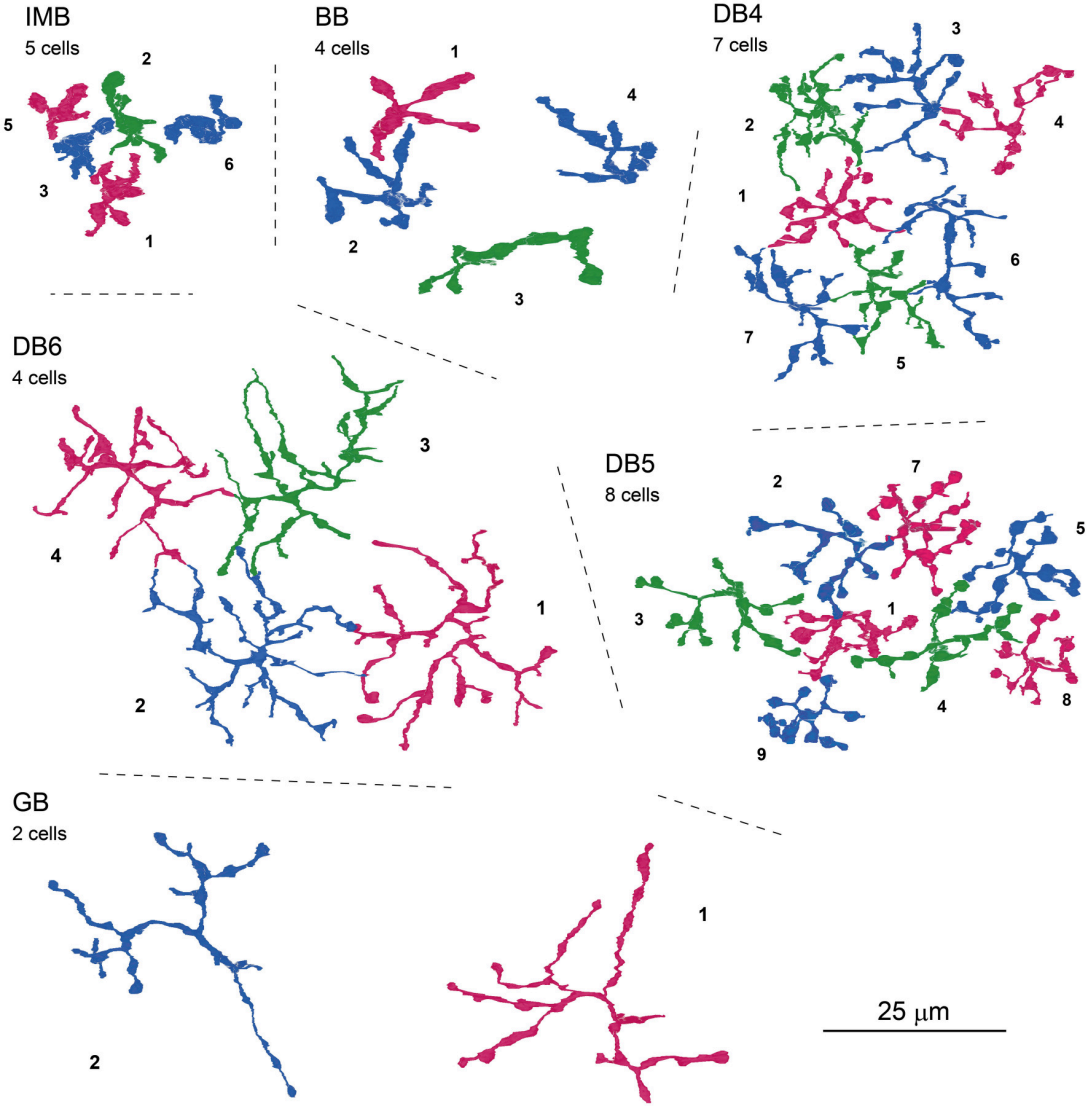
**Top-view profiles of axon terminal arbors of the ON bipolar cell types**. For each cell type, 2−8 neighboring cells of the same type are displayed with serial numbers. No other GB cells were found around GB-1 and GB-2. Cells are colored for clarity.

Next, we characterized the axonal terminal morphology of these ON bipolar cell types (three left-hand columns of Table 3). The stratification of the bipolar axon terminal was assessed by two variables, the terminal tip level (the axon-to-GCL distance) and the axon arbor thickness, using the side-view images of our 3D-reconstructed cells (Figure 1). The axon terminals of DB4, DB5, and GB cells were situated within stratum 3 of the IPL, those of IMB cells from the middle of stratum 3 to the border between stratum 4 and 5, and DB6 and BB cells from stratum 4 to the upper half of stratum 5. The DB6 axon arbor was 2-fold thicker than the GB axon arbor. Between these upper and lower limits, IMB and BB cells had the same mean thickness, which was slightly greater than those of DB4 and DB5 cells. We also measured the axon arbor area using the top view of the axon terminals (Figure 8). IMB cells had the narrowest and GB cells the widest axon arbor area, with a difference of about 15-fold. The axon arbor areas of BB and DB5 cells were roughly 3-fold larger than those of IMB cells, those of DB4 cells were 4-fold larger, and those of DB6 cells 10-fold wider than those of IMB cells.

**Table 3:** **Morphology and synaptic connectivity of ON bipolar axon terminals with ganglion cells**.

**Bipolar cell type (n)^a^**	**Distance between axon and the GCL (μm)**	**Axon arbor thickness (μm)**	**Axon arbor area (μm^2^)**	**Number of ribbons**	**Number of PG outputs per BC (*n* = 5)^b^**	**Number of MG outputs per BC (*n* = 25)^c^**	**Number of BC inputs per MG (*n* = 5)^d^**
IMB (5)	6.0 ± 0.8	9.7 ± 2.3	67.9 ± 15.4	85.0 ± 9.0	14.6 ± 3.2	73.8 ± 6.9	191.8 ± 21.3
BB (4)	3.3 ± 1.0	9.7 ± 1.3	198.7 ± 36.5	59.0 ± 7.5	0.0	1.8 ± 1.7	1.2 ± 1.6
DB4 (7)	11.1 ± 1.0	8.0 ± 1.4	246.7 ± 56.7	97.3 ± 3.8	25.0 ± 5.6	14.7 ± 6.6	10.0 ± 7.0
DB5 (8)	11.4 ± 0.8	7.5 ± 2.8	189.3 ± 50.2	67.8 ± 11.0	12.0 ± 4.0	4.8 ± 3.5	4.0 ± 1.6
DB6 (4)	2.3 ± 0.9	11.6 ± 3.1	647.7 ± 110.8	98.0 ± 8.7	2.0 ± 2.8	67.8 ± 8.8	30.2 ± 5.2
GB (2)	9.6 ± 2.3	6.5 ± 2.1	1061.5 ± 72.2	86.5 ± 14.8	27.5 ± 6.4	4.0 ± 1.4	0.4 ± 0.9
							237.6 ± 21.0^e^

a Number of sample bipolar cells of each type.

b Number of PG cells examined for the measurement of bipolar-PG contacts per bipolar cell.

c Number of MG cells examined for the measurement of bipolar-MG contacts per bipolar cell.

d Number of MG cells sampled for the measurement of bipolar-MG contacts per MG cell.

e The total of input synaptic contacts from all types of BCs.

**Figure 9:**
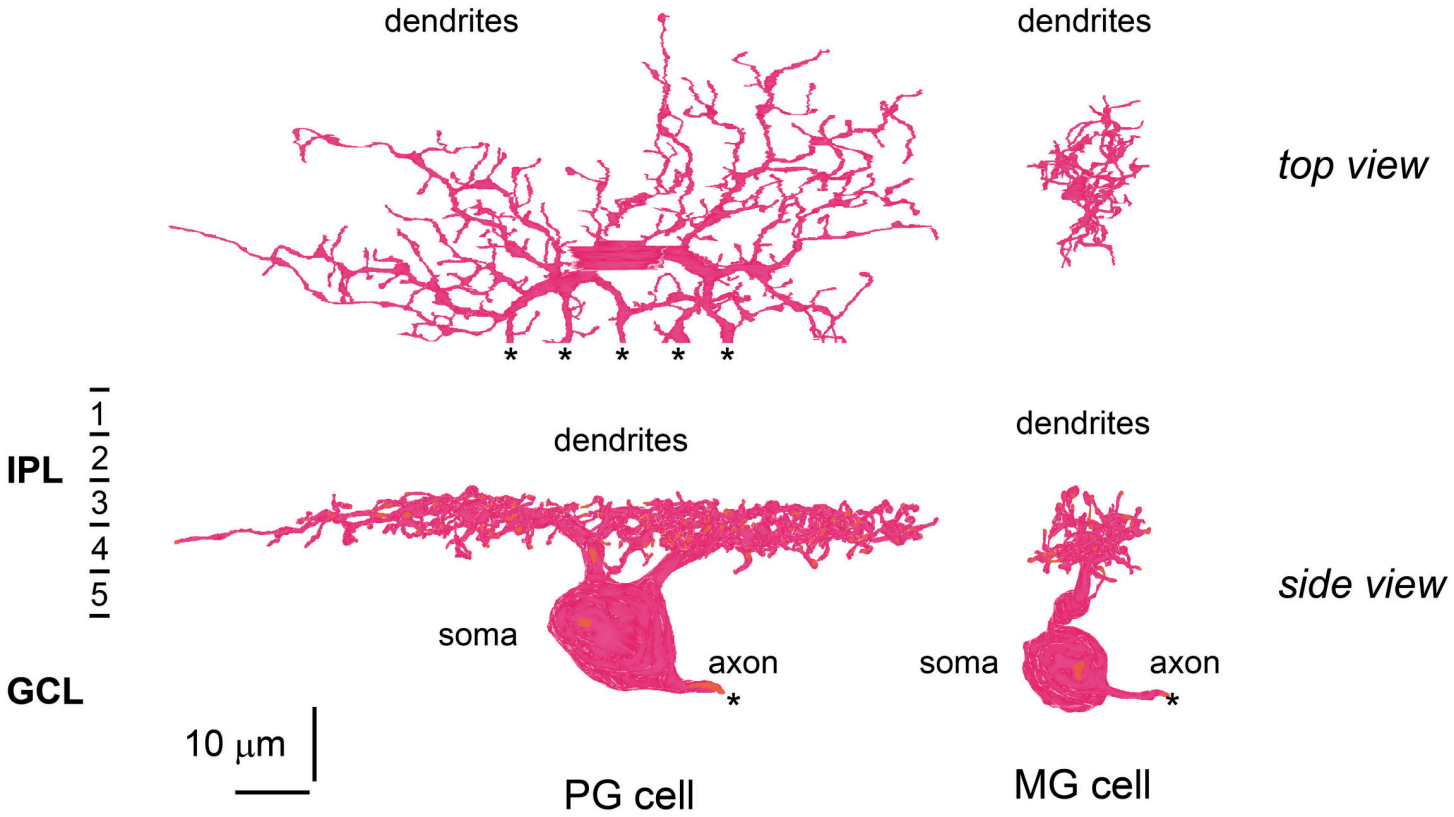
**Morphology and stratification of ON parasol and ON midget ganglion cells**. An ON parasol ganglion (PG) cell (left) and an ON midget ganglion (MG) cell (right) in top (upper) and side (lower) view. The dendrites of these ganglion cells extend into strata 3 and 4 of the IPL. Asterisks (^*^) indicate the end points of the series of electron micrographs.

**Figure 10:**
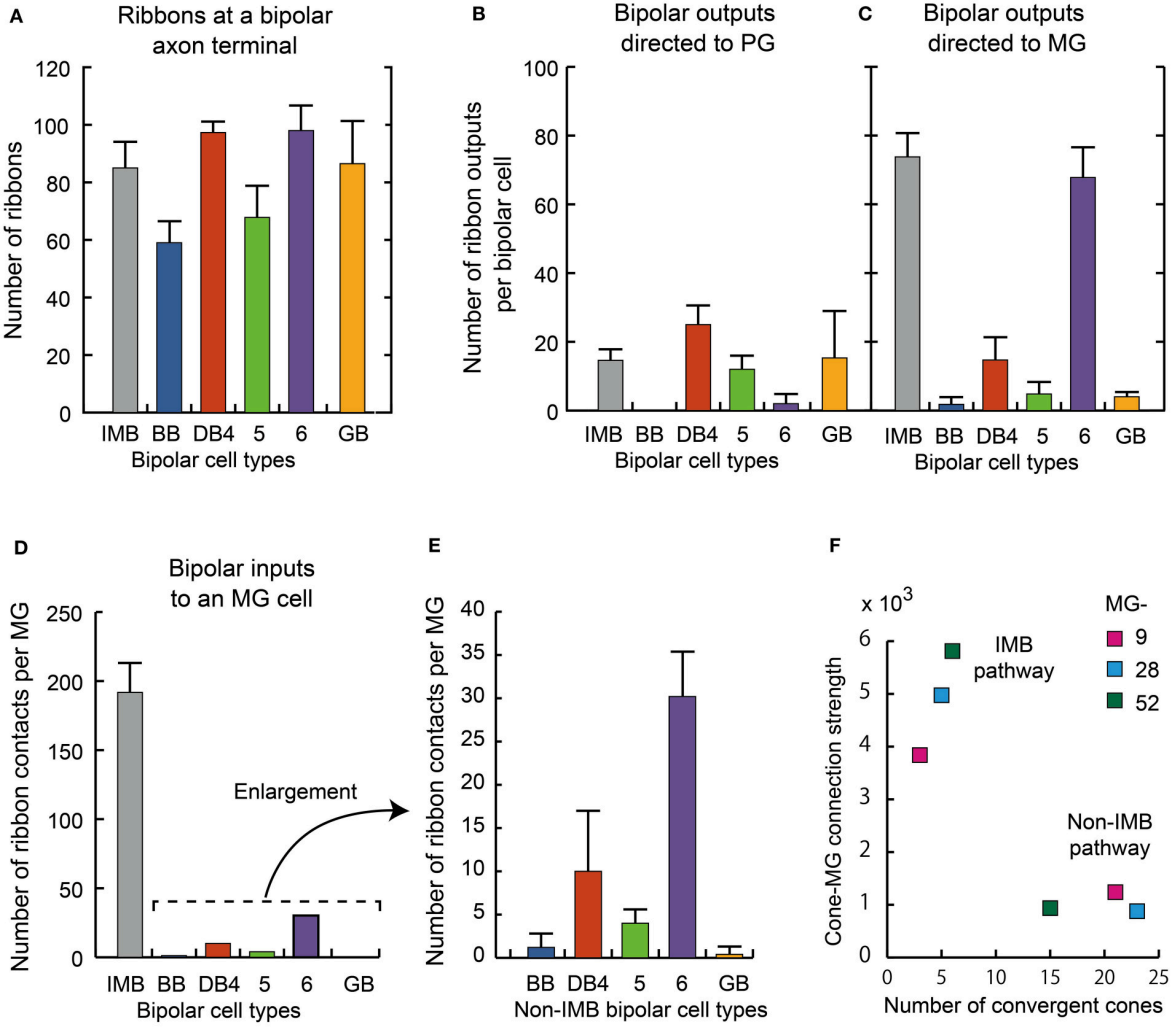
**Summary of axonal synapse measurement.(A)** The number of synaptic ribbons in the axon terminal for each type of ON bipolar cell. **(B,C)** The number of ribbon synaptic contacts with ON PG cell **(B)** and ON MG cells **(C)**. Data are mean ± SD for each type (number of cells): IMB (5), BB (4), DB4 (7), DB5 (8), DB6 (4), and GB (2). Thirty axon terminals were analyzed **(A**−**C)**. **(D)** Number of synaptic contacts from different types of bipolar cells to an MG cell. **(E)** Enlargement of the non-IMB part of **(D)**. **(F)** The relationship between the cone−MG connection strength expressed as the contact number product and the number of cones converging to the MG cell via the IMB and non-IMB (BB, DB4, 5, 6, GB) pathways. Three ganglion cells, MG-9, −28, and −52, were examined with their related bipolar and cone cells.

We further examined three additional ultrastructural parameters: the total number of axon synaptic ribbons, the number of output synaptic contacts directed to PG cells, and the number of synaptic contacts directed to MG cells (three middle columns of Table 3). The examples of PG and MG cell reconstruction are displayed in Figure 9. The number of axonal ribbons varied within the 2-fold range from 51 to 106 for all 30 cells examined and the mean number increased in the rank order BB < DB5 < IMB < GB < DB4 < DB6 (Figure 10A). The output of the ribbon synapses at the bipolar cell axon terminal was directed to various types of ganglion and amacrine cells. Here we focused on PG and MG cells. BB cells had no contacts with PG cells and few contacts with MG cells (Figures 10B,C). Types IMB, DB4, DB5, and GB all exhibited substantial numbers of contacts with PG cells; among them, type DB4 had the greatest number. In contrast, DB6 cells made few contacts with PG cells but, interestingly enough, a large number with MG cells. The mean number of contacts per bipolar cell for the DB6 type (68 contacts, 70% of the ribbons) was comparable to that of IMB cells (74 contacts, 87% of the ribbons), while the others made many fewer contacts with MG cells (15 by DB4, 5 by DB5, and 4 by GB). From the opposite point of view, MG cells are thought to mainly receive bipolar input through synapses with IMB and DB6 cells; therefore, we obtained the number of synaptic contacts for each bipolar type per MG cell (right-most column of Table 3 and Figures 10D,E). Consistent with the current notions of retinal organization, the MG cell received input predominantly through synapses with IMB cells (192 contacts), while the contribution of DB6 cells was modest (30 contacts). Nevertheless, DB6 cells had 3-fold more contacts with MG cells than DB4 cells (10 contacts) and 8-fold more than DB5 cells (4 contacts). BB and GB cells had negligible numbers of contacts with MG cells.

### Cluster analysis using axonal morphology and synaptic connectivity

**Figure 11:**
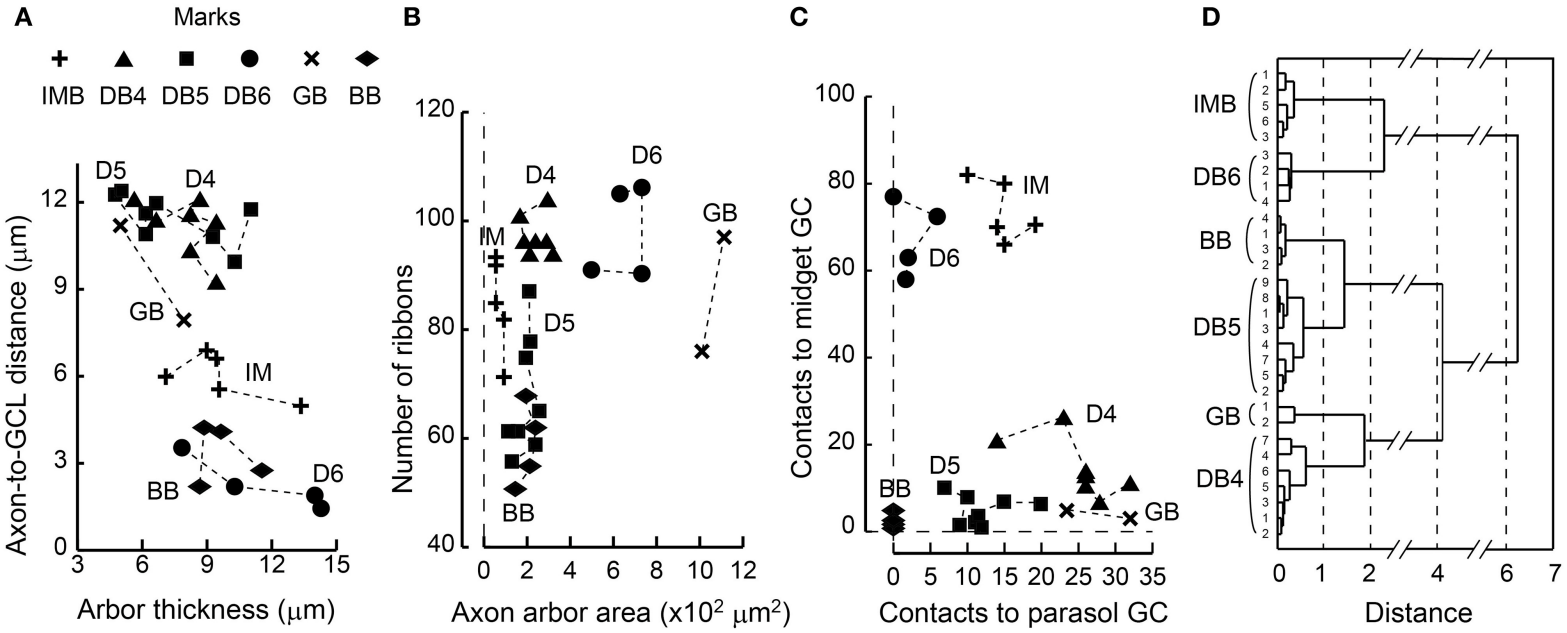
**Scatter plots of bipolar axon terminal counts and their clustering among the six ON bipolar cell types. (A)** The relationship between the distance from the axonal tip to the ganglion cell layer and the thickness of the axonal arbor. **(B)** The relationship between the number of synaptic ribbons and the top-view area of an axonal arbor. **(C)** The relationship between the number of bipolar output contacts directed to MG cells and the number directed to PG cells. **(D)** Dendrogram of cluster analysis (Ward's method) of 30 ON bipolar cells using the six variables plotted in **(A**−**C)**. All cases are uniquely clustered into six types.

We once more confirmed the classification of ON bipolar cells by cluster analysis based on a set of six features of axon terminal arbors. The discriminative power of each feature is easily grasped by the scatter plots (Figures 11A-C). The stratification level of the axon terminal as measured by the axon-to-GCL distance has been used by many authors (e.g., Ghosh et al., 2004) and is one of the most powerful discriminators of bipolar cell types. The stratification level separated all cells into three groups: (i) DB4, DB5, plus GB, (ii) IMB alone, and (iii) DB6 plus BB. In contrast, arbor thickness was poorly discriminative (Figure 11A). The axon arbor area ranked four groups in the increasing order IMB < DB4, DB5, and BB < DB6 < GB, clearly distinguishing IMB and GB from the others. The number of ribbons only divided types into the great group (IMB, DB4, DB6, and GB) and the small group (DB5, BB) (Figure 11B). The number of contacts to MG cells prominently separated IMB and DB6 from the others, while the number of contacts with PG cells roughly sorted all the cells into three groups: (i) DB4 plus GB, (ii) IMB plus DB5, and (iii) DB6 plus BB (Figure 11C). The integration of all these multivariate discriminations separated all 30 ON bipolar cells examined into six separate clusters (Figure 11D).

Because DB4 and DB5 cells looked similar in side-view (Figure 1), we applied Student's *t*-test (unpaired two-tailed) to the afore-mentioned six feature parameters (*df* = 14, 8 cells for each type) and furthermore to the number of convergent cones per bipolar cell (*df* = 11, 6 DB4 cells and 7 DB5 cells). *P*-values were 0.40 (stratification level), 0.70 (arbor thickness), 0.028^*^ (arbor area), 0.000004^*^ (ribbon number), 0.001^*^ (MG output), 0.0009^*^ (PG output), and 0.042^*^ (cone number). The two former morphological features rendered no statistical significance as expected from their appearance, but the five latter parameters gave significant (^*^) differences at the 0.05 level.

### Comparison of cone−bipolar−ganglion connections between M/L- and S-cones

BB and IMB cells work together to cover all cone pedicles, BB cells for S-cones and IMB cells for M/L-cones. By contrast, FMB cells as a single type cover all cone pedicles, some FMB cells for S-cones (Klug et al., 2003; Tsukamoto and Omi, 2015a) and the other FMB cells for M/L cones. A small portion of the S-cone output is also delivered to DB and GB cells. Subsequent to this cone-bipolar junction, M/L-cone and S-cone signal components are allocated to MG and PG cells in the next bipolar-ganglion junction. The outputs of ON DB plus GB and OFF DB cells are directed mainly to PG cells but also moderately to MG cells. Here, we compared ON and OFF systems in terms of the cone sampling strength and its S-cone contribution via cone−bipolar−MG routes, and besides surveyed the routes to PG cells.

In the present analysis of the ON system, three ON MG cells (MG-9, −28, and −52) were situated in the middle of our examination area and so allowed tracing back to all converging cones via intervening ON bipolar cells. We divided the ON bipolar routes into IMB and non-IMB (DB4, 5, 6, GB, and BB) pathways (Table 4A). The number of cones converging onto each ON MG cell was small via the IMB pathway (5 cones) but relatively large via the non-IMB pathway (20 cones). Conversely, the cone−MG connection strength was intense via the IMB pathway (4867) but moderate via the non-IMB pathway (1018) (Figure 10F). Among members of the non-IMB pathway, DB6 and DB4 were the two major contributors to the total contact number products. These MG cells (MG-9, −28, and −52) happened to lack any contact with GB cells and consequently the contact number product via GB cells was zero. This is the reason for the absence of the GB type in Table 4A. However, other MG cells in this series had one or two contacts with GB cells, about 0.4 contacts per MG cell as shown in the right-most column of Table 3. Although, the GB type had the greatest number of convergent cones among all ON bipolar types, GB cells were connected to too many MG cells to have a substantial effect on most.

**Table 4:** **Cone sampling of ON and OFF MG cells**.

**(A) ON MG (3 CELLS)**						
**Mean of MG-9, -28, and -52**	**Number of convergent cones**			**Sum of contact number products**		
	**Total**	**M/L-cones**	**S-cones**	**Total**	**M/L-cones**	**S-cones**
MB channel	5.0	5.0	0.0	4875	4875	0
DB channel	19.7	18.0	1.7	1018	978	40
DB4	12.0	11.7	0.3	431	426	5
DB5	9.7	8.3	1.3	82	76	5
DB6	9.0	8.3	0.7	492	476	16
BB	1.3	0.0	1.3	13	0	13
**(B) OFF MG (2 cells having no S-cone contacts via the MB channel)**						
**Mean of MG-3 and -6**	**Number of convergent cones**			**Sum of contact number products**		
	**Total**	**M/L-cones**	**S-cones**	**Total**	**M/L-cones**	**S-cones**
MB channel	4.0	4.0	0.0	5966	5966	0
DB channel	22.0	20.0	2.0	1458	1412	46
DB1	16.0	14.5	1.5	959	944	15
DB2	15.5	13.5	2.0	392	362	30
DB3a	4.5	4.0	0.5	30	29	1
DB3b	4.0	4.0	0.0	77	77	0
**(C) OFF MG (1 cell having S-cone contacts via the MB channel)**						
**MG-4 alone**	**Number of convergent cones**			**Sum of contact number products**		
	**Total**	**M/L-cone**	**S-cones**	**Total**	**M/L-cone**	**S-cones**
MB channel	4	3	1	5522	3524	1998
DB channel	23	21	2	1742	1688	54
DB1	15	13	2	874	831	43
DB2	12	11	1	414	411	3
DB3a	10	9	1	326	318	8
DB3b	11	11	0	128	128	0

While the IMB pathway did not mediate S-cone to MG cell transmission, the non-IMB pathway mediated a weak S-cone connection to MG cells. In fact, 97% of the synapses at the S-cone pedicle were directed to BB cells, the remaining 3% (1.2 contacts per cone) were shared by DB4, 5, 6, and GB cells (Figure 6E). This may indicate the scarceness of S-cone contribution to the DB and GB pathways. The percentages of the number of S-cone contacts per bipolar cell are obtained from Table 1 as DB4 (0.5%) < DB5 (2.2%) < DB6 (2.7%) < GB (6%). In addition to DB and GB cells, BB cells provide ON MG cells with the S-cone component (Figure 10C, Table 3). Consequently, the M/L- to S-cone ratio measured by the contact number product for the ON MG cell is 96%: 4% via the ON non-IMB pathway (Table 4A).

Likewise, we obtained the comparative values of the OFF system using our previous data set (Tsukamoto and Omi, 2015a). We divided the OFF bipolar routes into FMB and DB (DB1, 2, 3a, and 3b) pathways. The number of cones converging onto each OFF MG cell was small via the FMB pathway (4 cones) but relatively large via the DB pathway (22 or 23 cones). By contrast, the cone–MG connection strength was intense via the FMB pathway (5966–5522) but moderate via the DB pathway (1458–1742). Among members of the DB pathway, DB1 and DB2 cells were the two major contributors to the total contact number products (Tables 4B,C).

Two OFF MG cells (MG-3 and -6) had no S-cone connection via FMB cells (Table 4B), but one OFF MG cell (MG-4) had a strong S-cone connection via an FMB cell, and all OFF MG cells examined also had a weaker S-cone connection via the DB pathway (Table 4C). In fact, 58% of the basal contacts at the S-cone pedicle were directed to FMB cells and the remaining 42% (22.5 contacts per S-cone) were shared by DB1, 2, 3a, and 3b cells. Although, this value of 42% appears relatively large as compared to the ON system, the numbers of basal contacts with DB cells at the M/L-cone pedicles (77.3 contacts per M/L-cone) are relatively large and M/L-cones outnumbered S-cones by a factor of 9. Thus, the S-cone to M/L-cone contact ratio is 1: 31. This provides OFF DB cells with the percentages of the number of S-cone contacts per bipolar cell equivalent to those of ON DB cells as DB3a (1.6%) < DB1 (2.6%) < DB3b (3.4%) < DB2 (6%). Consequently, the M/L- to S-cone ratio measured by the contact number product for the OFF MG cell is 97%: 3% via the OFF DB pathway (Tables 4B,C).

A similar scarcity of S-cone signals was also found for PG cells. BB cells had no contact with ON PG cells. Although IMB cells made many contacts to ON PG cells, they made no contacts with S-cones. In contrast to IMB cells, about one-tenths of the FMB cells made many contacts with an S-cone. This is because about one-tenths of the cones were S-cones in this examination area and each S-cone contacted one FMB cell. However, the FMB cells as a whole had very few contacts with OFF PG cells: 1 contact per bipolar cell compared to 67 contacts per bipolar cell with OFF MG. Only one-tenths of this 1 contact is from S-cone. Therefore, the S-cone signals may be prevented from reaching OFF PG cells via the FMB pathway. ON DB plus GB cells made numerous contacts with ON PG cells and OFF DB cells with OFF PG cells. However, as afore-mentioned, the percentages of the number of S-cone contacts per bipolar cell ranged from 0.5 to 6% for both ON DB plus GB cells and OFF DB cells. Therefore, the total contact number products along the S-cone−bipolar−PG route must be considerably smaller than the M/L-cone−bipolar−PG route. The contributions of S-cones to PG cells must be as small as those to MG cells.

## Discussion

Identification of bipolar circuits at the ultrastructural level is one of the most direct ways to characterize signal processes in the retina. First, we examined the synaptic contacts of the ON (cone) bipolar dendrites with photoreceptors (M/L-cones, S-cones, and rods) and their positional classes (invaginating, TA, and NTA), and obtained the number of contacts per bipolar cell and cone for each type. Next, we examined the axonal synaptic contacts with PG and MG cells and an ON-OFF lateral amacrine cell and then estimated their number of contacts per bipolar or MG cell. In both cases, we performed a cluster analysis to confirm the validity of ON bipolar cell classification based on the differences in these parameters. Finally, we compared S-cone vs. M/L-cone sampling strength of MG cells by calculating the contact number products. We performed these analyses to investigate the possible correspondence between ON and OFF bipolar cell types.

### Basal contacts of ON bipolar cells

We observed many basal contacts (TA and NTA) of ON bipolar cell dendrites with cone pedicles (Figures 2B,E,G,I) and also with rod spherules (Figures 2H,I) in our examination area at 3 mm eccentricity. The percentages of the basal contacts per bipolar cell are obtained from Figure 6C as IMB (1%), BB (1%), DB5 (38%), DB4 (52%), DB6 (66%), and GB (82%). Hopkins and Boycott (1995, 1997) reported similar data (number of sample cells: 1~2) by the EM-Golgi method for DB4 (44%), DB5 (10%), and DB6 (30%) at 1.9–4.0 mm eccentricity.

Calkins et al. (1996) reconstructed two ON DB cells (not typed further) contacting 10 foveal cones (with about 20 ribbons at 0.50–0.54 mm eccentricity) and observed that only 3~4 contacts of each ON DB cell were invaginating. Because these ON DB cells each had 25 contacts, they suggested that the remaining 21~22 (about 85%) contacts were basal. Chun et al. (1996) also recognized a similar situation of foveal cones (with about 21 ribbons at 0.75 mm) that no enough number of the invaginating positions were available to accommodate the dendrites of ON DB cells. They further observed peripheral cones (with about 42 ribbons at 5–6 mm) and pointed out that there were enough central positions available for the invaginating dendrites of both IMB and ON DB cells. As afore-mentioned, our percentage data of the basal contacts per bipolar cell with perifoveal M/L cones (with about 31 ribbons at 3 mm) were 38% (DB5) < 52% (DB4) < 66% (DB6) (Figure 6C). These were intermediate between almost none in the periphery and about 85% of the ON DB cell contacts in the fovea.

The distinction between ON and OFF types does not depend on the invaginating or basal contacts but on which type of glutamate receptor is expressed on the bipolar cell dendrites, metabotropic mGluR6 or ionotropic AMPA/Kainate (for review, Wässle, 2008). Electrophysiological experiments in ground squirrel retinas (DeVries et al., 2006; Szmajda and DeVries, 2011) demonstrated that the glutamate transmitter released from the ribbon-associated active zone effectively reach postsynaptic receptors at remote basal contacts of OFF bipolar cells. The same glutamate is thought to be available to the basal contacts of ON bipolar cells. Bipolar cells may implement diffusional filtering by their dendrite positioning (Rao-Mirotznik et al., 1998; Sterling and Laughlin, 2015). The localization of mGluR6 in rhesus monkey retinas was examined by Vardi et al. (2000) with the immuno-EM method. Their main conclusion was that mGluR6 was not on the tip of the central element, but on its base at the mouth of the invagination, 400–800 nm from the release site. They presented an EM picture showing that mGluR6-immnoreactive dendrites formed basal contacts at NTA positions. Their findings may indicate that the ON bipolar cell dendrites located at NTA positions still sense the glutamate diffused from the active zone. Although, they did not report any basal contacts of mGluR6-immunoreactive dendrites with rod spherules, one could imagine that the probability of encountering such contacts on immuno-EM sample sections might be extremely small. These lines of evidence convince us that the basal contacts of ON bipolar cells are not accidental but functional.

### Correspondence between ON and OFF bipolar cell types

#### IMB and BB vs. FMB

IMB cells correspond with FMB cells as both are midget types. BB cells are classified as a unique type but similar to FMB cells in that all BB cells and some FMB cells contact S-cones. Both IMB and BB types were necessary to tile all the cone pedicles. Ninety-nine percent of the synaptic contacts of both IMB and BB cells with cones were of the invaginating type located closest to the synaptic ribbon zone. Likewise, 93% of FMB cone contacts were of the TA class, which was closest to the ribbon zone among the positional classes of OFF-specific basal cone contacts (Tsukamoto and Omi, 2015a). Using four dendritic properties for cluster analysis, we could not discriminate IMB from BB cells (Figures 7C). In particular, a BB cell contacting a single S-cone was morphologically similar to an IMB cell. These findings imply that IMB and BB types are homologous and both are counterparts of the FMB type.

#### DB6 vs. DB1

In only one of the ON DB types, DB6, were the major output synapses predominantly directed to MG cells. Seventy percent of the axonal ribbons of the DB6 cell were associated with the output synapses directed to ON MG cells. This is comparable to the ~60% of axonal ribbons of DB1 cells directed to OFF MG cells (Tsukamoto and Omi, 2015a). DB6 and DB1 axon terminals were in the lowest and highest zones of the IPL, strata 5 and 1, respectively. The number of convergent cones was lowest for DB6 among DB4, 5, 6, and GB. Similarly, the number of convergent cones was lowest for DB1 among DB1, 2, 3a, and 3b. Therefore, DB6 likely corresponds to DB1.

#### GB vs. DB3b

Only one ON bipolar type, GB, made synaptic contacts with rods as well as cones (5 rods and 12.5 cones per GB cell, *n* = 2). Similarly, DB3b is the only OFF bipolar type with such rod and cone contacts (4.0 rods and 9.0 cones per DB3b cell, *n* = 4). The rod synapses of GB cells were not located at the invaginating position but at the rod spherule base. The GB cell had the most abundant NTA contacts and the fewest invaginating contacts among ON bipolar types. Also, the GB cell had the smallest number (4) of contacts directed to ON MG cells but the largest number (28) of contacts directed to ON PG cells. Likewise, the type DB3b cell had the most abundant NTA contacts among OFF bipolar types. Moreover, the DB3b cell had the smallest number (1) of contacts directed to OFF MG cells but relatively many (14) contacts directed to OFF PG cells. These findings suggest that both GB and DB3b cells may function cooperatively to convey mixed rod and cone signals to PG cells at relatively slow speed compared to other cone bipolar pathways. Nevertheless, when both GB and DB3b cells carry rod signals via direct contacts with rod spherules, they must be able to transmit more quickly than the rod−rod bipolar−AII amacrine indirect ON and OFF pathways. Our observations provide anatomical evidence for the insensitive but fast rod pathways observed in psychophysical experiments of achromatic observers lacking functioning cones (Stockman et al., 1991, 1995; Sharpe and Stockman, 1999).

As afore-mentioned, GB cells contacted rods as well as cones. The sampled cones of GB cells were sporadically distributed, where an S-cone was not excluded (Figure 5). The cone sampling strengths of GB cells were very small. Therefore, it is unlikely that GB cells convey reliable chromatic signals.

#### DB4 vs. DB2 and DB5 vs. DB3a

Initially we compared our classification of DB4 and DB5 types based on morphological features to the original work by Boycott and Wässle (1991). In their data, DB4 cells stratify between 44 and 47 μm from the cone pedicles and DB5 cells between 46 and 48 μm. The side-view of their DB4 and DB5 drawings (their Figure 4) shows that the flat axon arbor of a DB5 cell is at the same depth as the lower part of the axon arbor of a DB4 cell. Someone may have the impression that the stratification level of the DB5 axon terminal is deeper than that of the DB4, indeed so reviewed in later years (Grünert et al., 1994; Wässle, 2004; Joo et al., 2011). In our data set, however, all DB4 and DB5 cells stratify at the same level of the IPL, in stratum 3 (Figure 1). Nevertheless, our data used for classification agree with their data on three other features: (a) DB5 cells have distinctly varicose processes (their Figure 8; our Figure 8); (b) A DB4 cell contacts more cones than a DB5 cell (their Table 1; our Table 1); and (c) The axon arbor area of a DB4 cell is greater than that of a DB5 cell (their Table 1; our Table 3).

In the ON system, DB4 and DB5 cells had similar light microscopic appearance but several distinct synaptic architectural properties at the EM level. The dendrites of DB4 cells had fewer invaginating than non-invaginating (TA + NTA) contacts (48%:52%), but this relation was reversed (62%:38%) in DB5 dendrites. Likewise, in the OFF system where TAs were closest to the ribbon zone, there were fewer TA contacts than NTA contacts (30%:70%) in DB2 dendrites but this relation was reversed (81%:19%) in DB3a dendrites. In the axon terminals, the number of synaptic ribbons was greater in DB4 than DB5 (97:67) and also in DB2 compared to DB3a (133:77). Given these parallels, it is likely that DB4 corresponds to DB2 and DB5 to DB3a. Furthermore, the DB4−DB2 correspondence was confirmed by the connectivity with a criterion neuron; both DB4 and DB2 cells had numerous reciprocal synapses with an ON-OFF lateral amacrine cell.

### Relationship with polyak's work

Polyak (1941) described numerous distinct bipolar cells (termed *d, e, f, g*, and *h*) in the macaque and chimpanzee retina stained according to the Golgi method. Therefore, we asked whether any of these previously observed and described cells corresponded with any of the 12 types listed here. The *d*–variety was named the “mop” bipolar cell after its shape (Polyak, 1941). Polyak recognized the mop bipolar cell to be the rod bipolar cell identified by Cajal (1893). Unambiguously, this type is the same as the RB type in our observation. The *e* variety, also called the “brush” bipolar, shares several common properties with the DB4, DB5, and DB6 types, particularly the brush-like dendritic arborization and the relatively deep axonal stratification. The *f* variety, also called the “flat” bipolar, is very similar to the DB1, DB2, DB3a, and DB3b types in the shared flatly extended dendritic arborization and the relatively shallow axonal stratification. In particular, Polyak emphasized that some of the flat bipolar cells had contacts with cone pedicles and rod spherules. These “common rod and cone” bipolars of the *f* variety may correspond to the DB3b type, which was shown to have basal contacts with both cones and rods at the EM level (Tsukamoto and Omi, 2014). The *g* variety shows a uniquely wide and sparse dendritic arborization. Polyak also stated that they were “found in few localities only.” Therefore, we are convinced that the *g* variety is the GB type. The *h* variety, also called the “midget” bipolar, evidently includes two groups distinguished by different stratification levels of the axon terminal. The one group terminating in the upper zone of the IPL may correspond to the FMB type and the other group terminating in the lower zone may correspond to the IMB type. Thus, all the bipolar cells that Polyak (1941) described are included in the catalog of this study. However, we could not find any BB-like cells in Polyak's figures and Polyak did not mention the BB cell. However, we cannot entirely exclude the possibility that the *h* variety included the BB cell contacting a single S-cone.

### S-cone contribution to MG and PG cells

The S-cone to M/L-cone ratio is approximately 1: 9 in the present examination area. However, every ganglion cell does not have about 10% of S-cone signal component. The contact number analysis shows that the S-cone component was unevenly delivered by three steps through the cone-bipolar-ganglion routes. First, an S-cone had 42 contacts whereas an M/L-cone had 52 contacts on average (Table 2), thus the S-cone component decreased by a factor of 0.8. Second, via cone-bipolar synapses, the outputs of an S-cone were predominantly targeted up to four BB cells or an FMB cell. As a result, most of the S-cone component was carried by BB cells for ON signal and by about one-tenths of the FMB cells for OFF signal. IMB cells have no S-cone component. For DB plus GB cells, the percentages of S-cone contacts per bipolar cell were ranged from 0.5 to 6%. Third, the major stream of the S-cone component was directed to small bistratified ganglion cells for ON signal and some OFF MG cells for OFF signal. The minor stream of S-cone component was directed via ON DB plus GB cells and OFF DB cells to MG and PG cells. For ON system, in addition, BB cells slightly contributed to ON MG cells but not to ON PG cells (Figure 10). Taken together, MG and PG cells form M/L-cone dominant pathways except some OFF MG cells.

Lee et al. (2004) reported that most DB6 cells contacted S-cones in macaque retina as revealed by immunofluorescence, with a M/L-cone: S-cone contact number ratio of 8: 1. They suggested that the DB cell may carry a “Yellow-ON” signal as the major spectral component. We agree with this idea based on our data that the contact number product ratio of M/L-cone: S-cone is even larger at 30: 1 for DB6 (Table 4). Furthermore, we may extend the same idea of “Yellow” signal as the major spectral component to the ON DB plus GB and the OFF DB pathways. Our data may provide a structural basis for the high-resolution physiological results of Field et al. (2010), who showed there was strong S-cone input to some OFF MG cells and a scarcity of S-cone input to most MG cells and all PG cells. In general, with the exception of the just-mentioned OFF MG cells connected to S-cones, the weakness of the S-cone connection is consistent with the physiological data of Sun et al. (2006), who showed that S-cone inputs to MG-parvocellular and PG-magnocellular cells were negligible. Tailby et al. (2008) also showed that the majority of MG-parvocellular and PG-magnocellular cells received no detectable input from S-cones in the LGN. These studies may strengthen the notion that all bipolar cell types that connect the cones laden with neural images to the ganglion cell outputs may limit visual perception by higher centers.

## Author contributions

The authors had full access to all the data in the study and take full responsibility for the integrity and the accuracy of the data analysis. YT designed this study, took micrographs, acquired data, interpreted results, and wrote the manuscript. NO took micrographs, acquired data, and checked the manuscript.

### Conflict of interest statement

The authors declare that the research was conducted in the absence of any commercial or financial relationships that could be construed as a potential conflict of interest.
